# Open-label study of long-term administration of dotinurad in Japanese hyperuricemic patients with or without gout

**DOI:** 10.1007/s10157-019-01831-5

**Published:** 2019-12-26

**Authors:** Tatsuo Hosoya, Masahiko Fushimi, Daisuke Okui, Tomomitsu Sasaki, Tetsuo Ohashi

**Affiliations:** 1 Jikei University School of Medicine, 3-25-8, Nishi-Shimbashi, Minato-ku, Tokyo, 105-8461 Japan; 2 Development Department, Medical R&D Division, Fuji Yakuhin Co., Ltd, 4-383, Sakuragi-cho, Omiya-ku, Saitama-shi, Saitama, 330-9508 Japan

**Keywords:** Hyperuricemia, Gout, Selective urate reabsorption inhibitor, URAT1 inhibitor, Dotinurad, FYU-981

## Abstract

**Background:**

Dotinurad is a novel selective urate reabsorption inhibitor (SURI) which reduces serum uric acid levels by selectively inhibiting urate transporter 1 (URAT1). This study was intended to verify the efficacy and safety of dotinurad following treatment for 34 or 58 weeks in hyperuricemic patients with or without gout.

**Methods:**

This long-term study had an open-label design with dose escalation. The dose of dotinurad started at 0.5 mg/day and was increased progressively to 2 mg/day. If the serum uric acid level of patients did not reach ≤ 6 mg/dL at week 14, the dose was increased to 4 mg/day. The primary endpoint was the percent change in serum uric acid level from the baseline to each visit.

**Results:**

At a dose of 2 mg, serum uric acid levels at week 34 and 58 were reduced from the baseline by 46.73% and 47.17%, respectively; at 4 mg, the respective values were 54.92% and 57.35%. At week 34 and 58, the percentages of patients achieving a serum uric acid levels ≤ 6.0 mg/dL with 2-mg dose were 89.11% and 91.30%, respectively; with 4 mg, the respective rates were 97.50% and 100.00%. In addition, the incidences of adverse events and adverse drug reactions were 65.2% and 21.8%, respectively.

**Conclusion:**

Dotinurad at doses of 2–4-mg sufficiently reduced serum uric acid levels in hyperuricemic patients with or without gout, and its efficacy and safety were verified for long-term administration.

ClinicalTrials.gov Identifier: NCT03006445

## Introduction

In Japan, hyperuricemia is defined as a serum uric acid level > 7.0 mg/dL; this causes urate crystal deposition diseases such as gouty arthritis and gouty tophus [1]. The number of hyperuricemic patients with or without gout has increased, such that the prevalence of gout in Japanese men older than 30 years is > 1% [1]. Recently, hyperuricemia has been reported to be associated with renal impairment and identified as a risk factor for renal failure [2, 3].

In the Japanese guidelines for the management of hyperuricemia and gout, the need for pharmacological therapy in hyperuricemic patients is determined by the serum uric acid level, with a target of maintaining the serum uric acid level ≤ 6.0 mg/dL, in consideration of the solubility of urate crystals [1]. It has been reported that when patients with gout who have maintained a serum uric acid level of less than 6.0 mg/dL for approximately 5 years discontinue treatment, gouty arthritis recurs within 4 years in approximately 30% of patients [4]. Therefore, long periods of treatment for hyperuricemia and gout may be required to control serum uric acid levels, using both lifestyle modification and antihyperuricemic drugs.

In Japan, xanthine oxidase inhibitors (XOIs) such as allopurinol, febuxostat, and topiroxostat, and uricosuric drugs such as benzbromarone, probenecid, and bucolome are used for hyperuricemic treatment. These drugs sometimes induce adverse drug reactions (ADRs) including hepatic dysfunction. Additionally, lesinurad, a selective urate reabsorption inhibitor (SURI) that was approved in the United States and European countries, acute kidney injury was reported as an ADR with high-dose monotherapy in a clinical study [5]. Under these circumstances, we believe that additional options are required to develop more effective and safe treatments in hyperuricemic patients with or without gout. Dotinurad is a novel SURI that reduces serum uric acid levels by selectively inhibiting URAT1 [6].

To date, studies of the clinical pharmacology of the drug have confirmed that no dose adjustment is required in elderly patients or in those with mild or moderate renal dysfunction [NCT02344875, NCT02347046]. In addition, dotinurad was shown to be extremely effective and safe by comparing with placebo in the phase II studies [NCT02344862, NCT02416167].

Following on these findings, the efficacy and safety of dotinurad were examined following treatment for 34 or 58 weeks in hyperuricemic patients with or without gout, in expectation of the need for long-term treatment.

## Methods

### Study design

This phase 3, multicenter, open-label, dose-escalation study was conducted for 34- or 58-weeks treatment at 26 clinical institutions in Japan.

### Inclusion and exclusion criteria

The inclusion criteria were a serum uric acid level during the run-in period ≥ 7.0 mg/dL (patients with a history of gouty arthritis or gouty tophi), ≥ 8.0 mg/dL (patients with asymptomatic hyperuricemia who were diagnosed with or treated for hypertension, diabetes mellitus, and/or metabolic syndrome), or ≥ 9.0 mg/dL (patients with asymptomatic hyperuricemia without the aforementioned complications); all were Japanese outpatients aged 20 years or older on the day that written informed consent obtained for participation in this study. The serum uric acid levels criteria followed the Japanese management guidelines [1].

The exclusion criteria were as follows: gouty arthritis that had not become asymptomatic within the 2 weeks before the day of randomization; the presence of disorders that might have caused secondary hyperuricemia; hemoglobin A1c (HbA1c; NGSP) ≥ 8.4%; use of drugs with the potential to affect the outcome of this study during the 2 weeks before the first day of the run-in period leading up to randomization; hyperuricemia classified as “overproduction type” or an indeterminate; complications of a serious cardiac disorder; a history of myocardial infarction and/or an anginal attack within a year; complications or a history of cancer (in the 5 years before informed consent was obtained); complications of hepatic impairment or aspartate aminotransferase (AST) and/or alanine aminotransferase (ALT) ≥ 100 U/L; complications of a renal calculus or clinical manifestations raising suspicion of a urinary calculus (e.g., hematuria and back pain); estimated glomerular filtration rate (eGFR) < 30 mL/min/1.73 m^2^; blood pressure ≥ 180-mmHg systolic and/or ≥ 110-mmHg diastolic; complications of stroke; a history of drug allergy; and presence of any other clinically significant medical conditions that could potentially preclude participation in this study. If patients had been treated with any antihyperuricemic or drugs affecting the serum uric acid level prior to their enrolment in this study, they were entered in this study only after a wash-out period of 2–4 weeks.

### Treatment

**Fig. 1:**
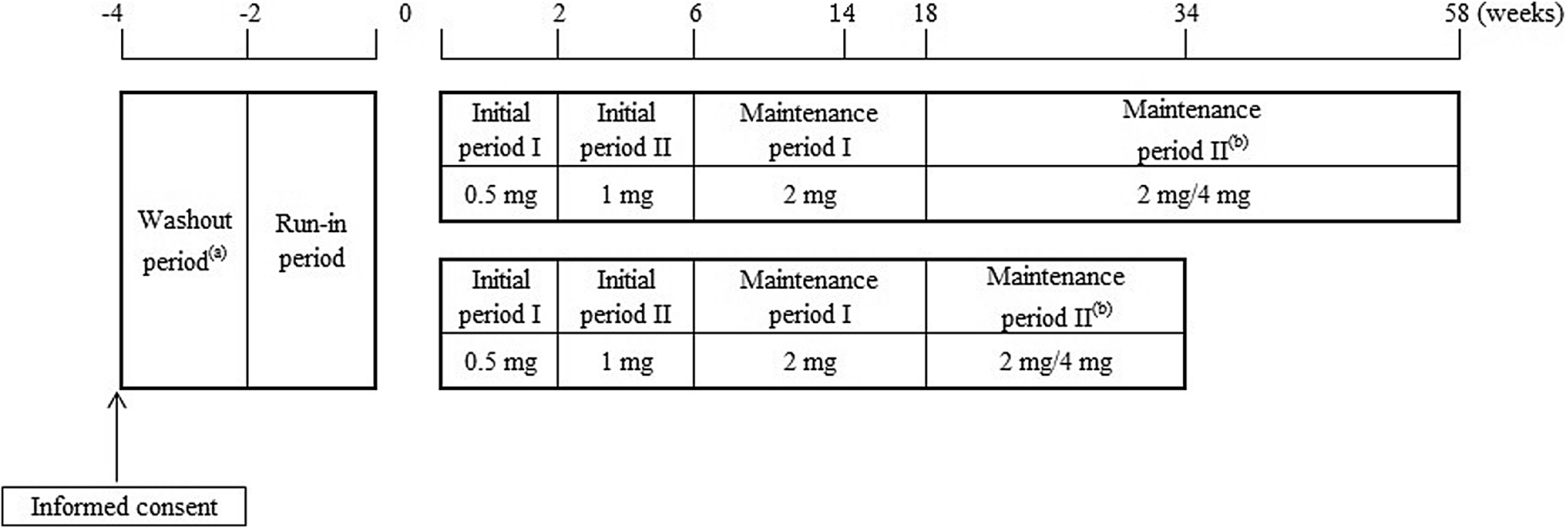
Dosing schedule. **a** Patients who had been treated with uric acid lowering drugs or treatment affecting the serum uric acid level were subjected to the wash-out period. **b** When a patient failed to achieve a serum uric acid ≤ 6.0 mg/dL at week 14, the dose was increased to 4 mg after week 18

Figure 1 shows the dosing protocol. In this study, the treatment period was 34 or 58 weeks. The first 120 patients assigned to the study were treated for 58 weeks and the remainder were treated for 34 weeks. Dotinurad was administered orally once daily. To avoid gouty arthritis due to a rapid decrease in serum uric acid levels, we adopted the approach of gradual dose titration [7]. The initial dose was dotinurad 0.5 mg/day for 2 weeks followed by 1 mg/day for 4 weeks. For the maintenance dose, dotinurad 2 mg/day was administered from week 6 to week 34 or 58. If the serum uric acid level exceeded 6.0 mg/dL at week 14, then the dose of dotinurad was increased to 4 mg/day, starting on the next visit (week 18) and continuing this treatment until week 34 or 58 had elapsed.

To minimize the risk of urinary calculi associated with increased urinary uric acid excretion, a urinary alkalization drug (e.g., citrate) was given with the dotinurad in the following circumstances: (1) a history of urolithiasis, (2) urine pH < 6.0 (from obtaining of informed consent to the end of this study), and (3) needs for the therapy at an investigator’s discretion. The addition of colchicine was not allowed throughout the study period, as this might have influenced the incidence of gouty arthritis as a safety endpoint.

### Classification of hyperuricemia

To confirm the type of hyperuricemia, hyperuricemia was classified based on measurement of uric acid in a 60-min urine collection obtained during the run-in period. Hyperuricemia was classified into the following four types: (i) patients with urinary extraction of uric acid [*E*_UA_ (mg/kg/h)] > 0.51 and uric acid clearance [*C*_UA_ (mL/min/1.73 m^2^)] ≥ 7.3 were defined as “overproduction type”, (ii) patients with *E*_UA_ < 0.48 or *C*_UA_ < 7.3 were defined as “underexcretion type”, (iii) patients with *E*_UA_ > 0.51 and *C*_UA_ < 7.3 were defined as “combined type”, and (iv) patients with 0.48 ≤ *E*_UA_ ≤ 0.51 and *C*_UA_ ≥ 7.3 were defined as “normal type”. The patients classified as “overproduction type” were excluded from this study because of the potential for urinary calculus formation. In this study, hyperuricemia was classified according to the second edition of the Japanese management guidelines, the latest version at the start of the study [8].

### Efficacy endpoints

The primary efficacy endpoint was the percent change in serum uric acid level from the baseline to each visit. The secondary efficacy endpoints were the percentage of patients achieving a serum uric acid level ≤ 6.0 mg/dL at each visit, the serum uric acid level at each visit, the change in eGFR, and in homeostatic model for assessment of insulin resistance (HOMA-IR) at the final visit.

### Safety evaluations

Adverse events (AEs) and safety assessments were conducted by clinical investigators based on vital signs, 12-lead electrocardiography, abdominal ultrasound and plain abdominal radiography, clinical laboratory tests, and clinical examinations throughout this study. AEs were classified according to the system organ class and preferred term (MedDRA version 21.0; Japanese Maintenance Organization, Tokyo, Japan) and were evaluated in terms of their potential causality with the study drug, severity, and seriousness. AEs judged to be related to the study drug were defined as adverse drug reactions (ADRs).

### Statistical analyses

The target sample size in this study was 330 patients for the 28-week maintenance period and 120 patients for the 52-week maintenance period based on the sample size and treatment period required to evaluate the safety of a novel drug that is assumed to be administered for a long-term period in the ICH-E1 guideline, and in consideration of patients who might discontinue the study.

The efficacy analysis was performed using the full analysis set (FAS), consisting of patients who received at least one dose of the study drug and had at least one efficacy endpoint evaluated after administration of the study drug. For the primary endpoint, summary statistics and two-sided 95% confidence intervals (CIs) of the rate of reduction (percent change) in serum uric acid levels from baseline were calculated for each visit across all dotinurad doses, as well as by dose at the completion of treatment (2 mg, 4 mg) using a one-sample *t* test. Additionally, summary statistics and two-sided 95% CIs were calculated for the reduction in serum uric acid levels by sub-group, based on eGFR. Taking the percentage of patients achieving a serum uric acid level ≤ 6.0 mg/dL at each visit as the secondary endpoint, the frequency was determined for all dotinurad doses and by dose at the completion of treatment (2 mg, 4 mg), to calculate the two-sided 95% CI of the percentage of success. Summary statistics and two-sided 95% CI of serum uric acid levels were calculated at each time point for all dotinurad doses and by dose at completion of treatment (2 mg, 4 mg). For eGFR and HOMA-IR, the changes from the baseline to week 34, week 58, and final visit were calculated for each dose (2 mg, 4 mg) and total at the last visit. Furthermore, the summary statistics and two-sided 95% CI were calculated and paired *t *test was conducted.

The safety analysis was performed using the safety population (SP), consisting of subjects who received at least one dose of the study drug and who had evaluable information on safety after administration of the study drug. The number of cases with, and the incidence and number of events involving AEs and ADRs were collected and calculated. Additionally, eGFR data were collected and calculated by sub-group in a similar manner.

Statistical analysis was performed using SAS software, version 9.3 (SAS Institute, Cary, NC, USA). The significance level of the test was 5% (two-sided).

## Results

### Patient flowcharts and baseline characteristics

**Fig. 2:**
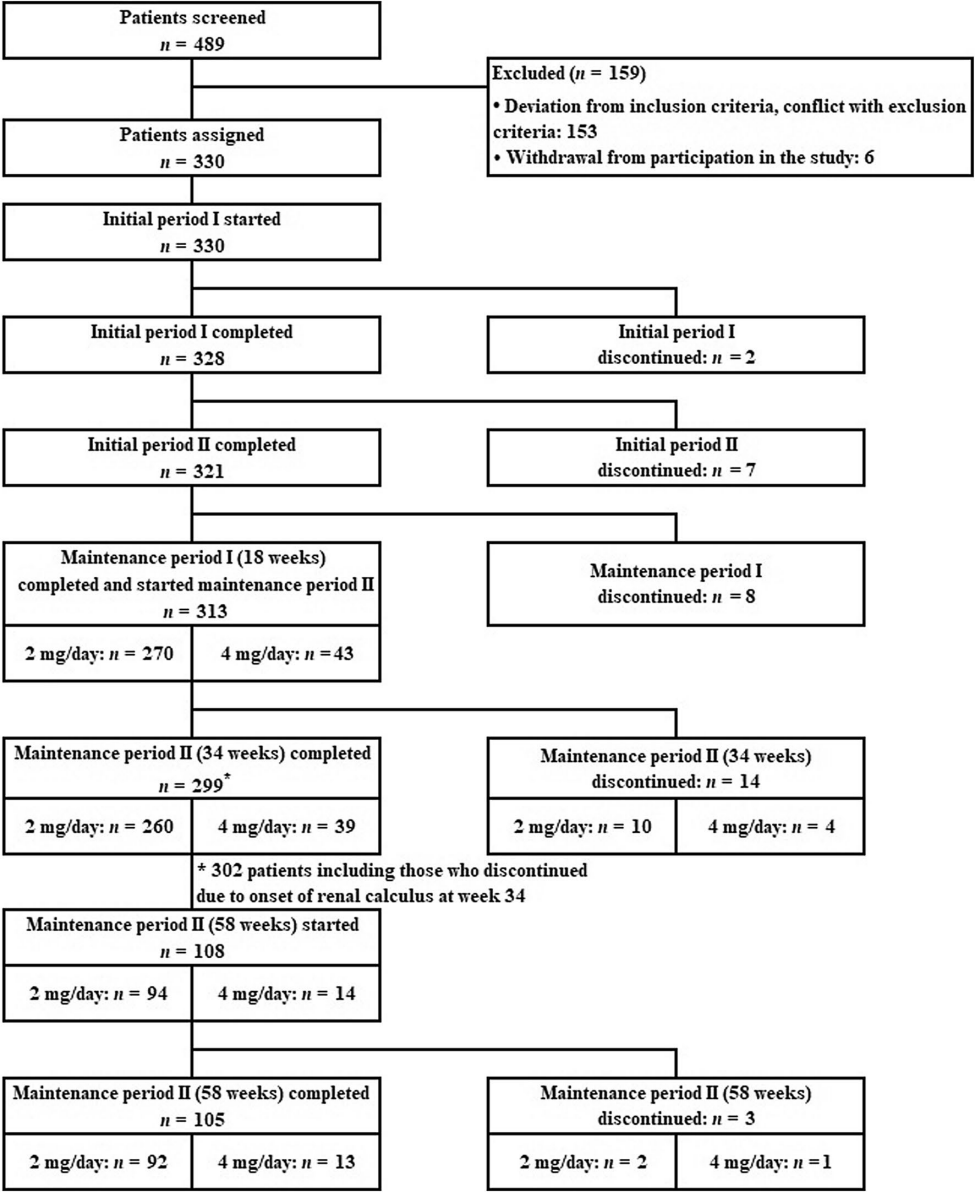
Flow diagram of patients in this study

Figure 2 summarizes the flow diagram of the study protocol. In this study, informed consent was obtained from 489 patients and the 330 patients confirmed to be eligible started administration of dotinurad. Subsequently, 34 patients withdrew from the study. At week 18, a total of 313 patients started maintenance period II and 270 patients continued to receive treatment with dotinurad 2 mg/day; 43 patients required dose escalation to 4 mg. Additionally, 299 patients completed 34 weeks of treatment (excluding three patients who discontinued treatment due to a renal calculus at week 34) and 108 patients transitioned to the 58-week treatment period. Of these 108 patients, 105 patients completed 58 weeks of treatment. Table 1 summarizes patient characteristics.

**Table 1:** Baseline characteristics of patients enrolled

Characteristic		Overall (*n* = 326)	2 mg* (*n* = 276)	4 mg* (*n* = 43)
Sex	Male	324 (99.4)	274 (99.3)	43 (100.0)
	Female	2 (0.6)	2 (0.7)	0 (0.0)
Age (year)	Mean ± SD	53.9 ± 10.5	54.2 ± 10.4	51.3 ± 10.5
Height (cm)	Mean ± SD	170.18 ± 6.05	170.07 ± 6.02	170.86 ± 6.08
Weight (kg)	Mean ± SD	76.77 ± 12.37	76.01 ± 11.90	82.17 ± 14.12
Serum uric acid level (mg/dL)	Mean ± SD	8.79 ± 1.13	8.63 ± 1.03	9.76 ± 1.25
eGFR (mL/min/1.73 m^2^)	Mean ± SD	69.6 ± 13.2	69.7 ± 12.7	70.0 ± 15.7
HOMA-IR	Mean ± SD	1.77 ± 1.59	1.69 ± 1.39	2.32 ± 2.49
Medical history (hyperuricemia)	No	180 (55.2)	156 (56.5)	21 (48.8)
	Yes	146 (44.8)	120 (43.5)	22 (51.2)
History of gouty arthritis	No	55 (16.9)	49 (17.8)	6 (14.0)
	Yes	271 (83.1)	227 (82.2)	37 (86.0)
Gouty tophus	No	320 (98.2)	272 (98.6)	42 (97.7)
	Yes	6 (1.8)	4 (1.4)	1 (2.3)
Comorbidity**	No	42 (12.9)	38 (13.8)	3 (7.0)
	Yes	284 (87.1)	238 (86.2)	40 (93.0)
Concomitant drugs**	No	122 (37.4)	103 (37.3)	17 (39.5)
	Yes	204 (62.6)	173 (62.7)	26 (60.5)
Drinking habit	No	156 (47.9)	135 (48.9)	19 (44.2)
	Yes	170 (52.1)	141 (51.1)	24 (55.8)
History of urinary calculus	No	291 (89.3)	248 (89.9)	41 (95.3)
	Yes	35 (10.7)	28 (10.1)	2 (4.7)
Type of hyperuricemia	Underexcretion type	279 (85.6)	237 (85.9)	36 (83.7)
	Combined or normal type	47 (14.4)	39 (14.1)	7 (16.3)

Counting value/nominal scale is expressed as *n* (%)

*eGFR* estimated glomerular filtration rate, *HOMA-IR* homeostatic model assessment for insulin resistance

*Dose at completion of treatment (excluding patients who withdrew at a dose of 0.5 or 1 mg)

**The main comorbidities were hypertension, dyslipidemia, and diabetes, and concomitant drugs were antihypertensives, vasodilators, and dyslipidemia

### Efficacy

#### The primary efficacy endpoint

**Fig. 3:**
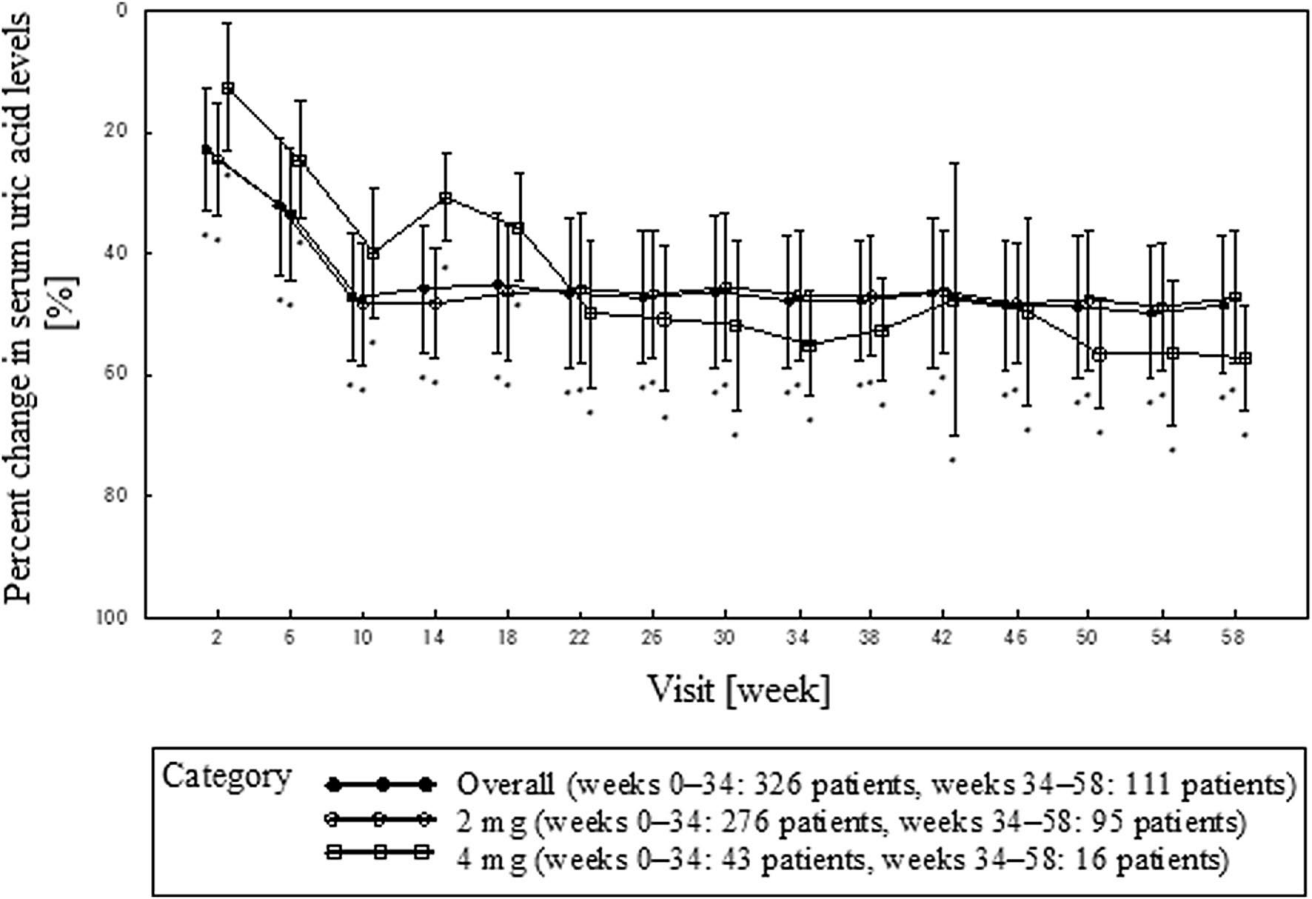
Percent change in serum uric acid levels from the baseline to each visit. Error bars indicates standard deviation.*P < 0.05

Table 2 and Fig. 3 show the percent change in serum uric acid levels from the baseline at week 34 and 58, and each visit, respectively. At week 34 and 58, the percent changes in serum uric acid levels (mean ± SD) from the baseline were as follows: 47.83% ± 10.85% and 48.43% ± 11.38% overall; 46.73% ± 10.77% and 47.17% ± 11.18% at a dose of 2 mg; and 54.92% ± 8.58% and 57.35% ± 8.73% at a dose of 4 mg. Significant differences were found at all time points in comparison to baseline (one-sample *t test*: *P* < 0.001). There was no difference in uric acid lowering effect between underexcretion and combined, normal type.

**Table 2:** Percent change in serum uric acid levels at week 34 and 58 by the type of hyperuricemia and all types

Type of hyperuricemia	Visit	Overall					2 mg					4 mg				
		*n*	Mean	±	SD	95% CI	*n*	Mean	±	SD	95% CI	*n*	Mean	±	SD	95% CI
All types	Week 34	297	47.83	±	10.85	46.59 to 49.07	257	46.73	±	10.77	45.41–48.05	40	54.92	±	8.58	52.18–57.67
	Week 58	105	48.43	±	11.38	46.23 to 50.64	92	47.17	±	11.18	44.86–49.49	13	57.35	±	8.73	52.07–62.62
Underexcretion type	Week 34	256	47.34	±	11.19	45.97 to 48.72	223	46.18	±	11.09	44.72 to 47.65	33	55.19	±	8.46	52.19 to 58.19
	Week 58	97	48.14	±	11.38	45.85 to 50.43	88	47.06	±	11.01	44.73 to 49.40	9	58.63	±	9.90	51.02 to 66.24
Combined type*	Week 34	34	51.39	±	8.04	48.58 to 54.19	27	50.79	±	7.63	47.78 to 53.81	7	53.67	±	9.75	44.65 to 62.69
	Week 58	7	52.98	±	12.11	41.77 to 64.18	3	51.01	±	19.73	2.00 to 100.2	4	54.45	±	5.22	46.14 to 62.76
Normal type*	Week 34	7	48.53	±	7.32	41.76 to 55.30	7	48.53	±	7.32	41.76 to 55.30	–	–	±	–	–
	Week 58	1	45.33	–	–	–	1	45.33	–	–	–	–	–	±	–	–

*Post hoc analysis

#### The secondary efficacy endpoint

**Fig. 4:**
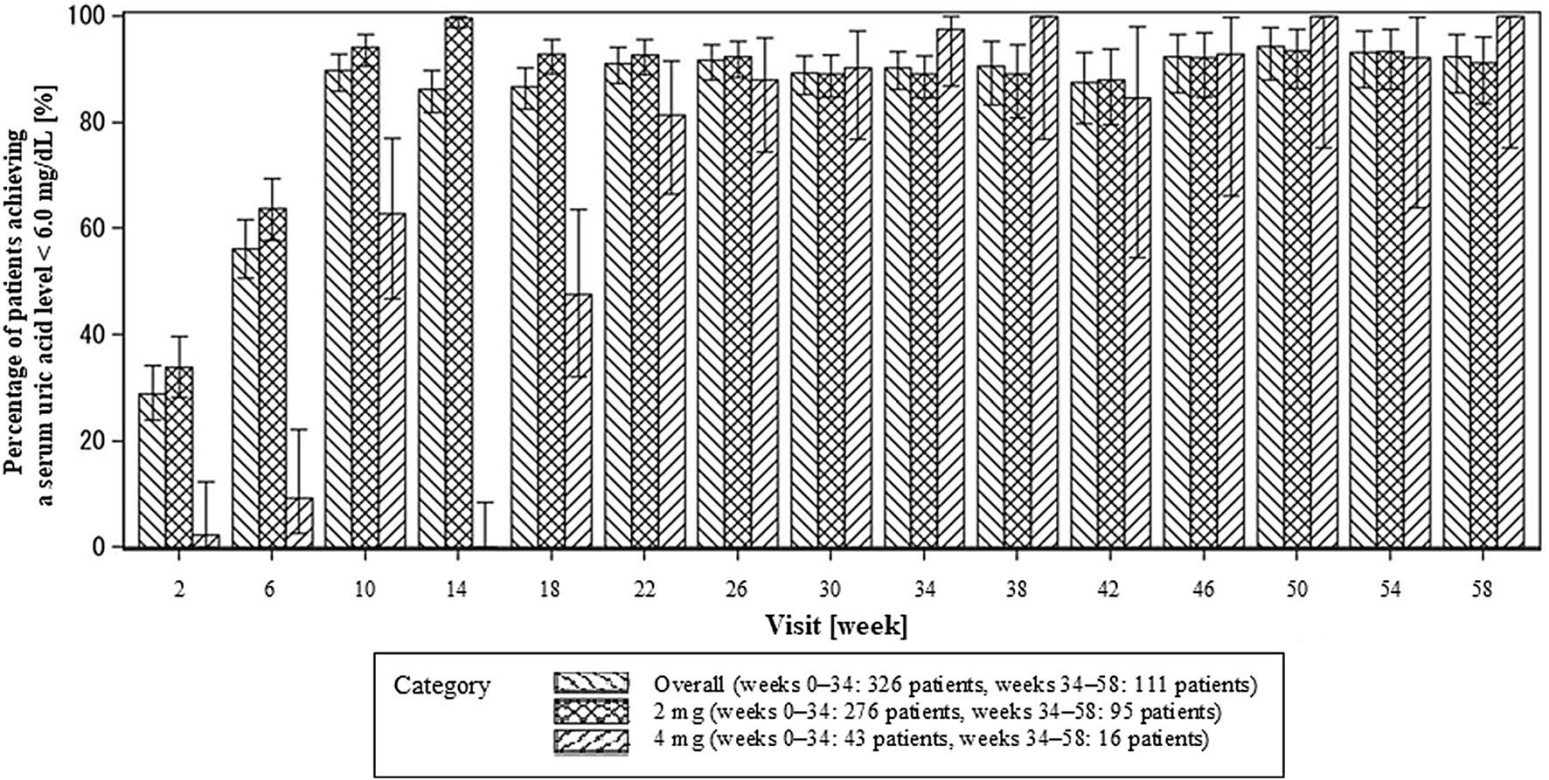
Percentage of patients achieving a serum uric acid level ≤ 6.0 mg/dL at each visit. Error bars indicates standard deviation

Figure 4 shows the percentage of patients achieving a serum uric acid level ≤ 6.0 mg/dL at each visit. The percentages of patients achieving a serum uric acid level ≤ 6.0 mg/dL at week 34 and 58 were as follows: 90.24% and 92.38% overall; 89.11% and 91.30% with 2 mg, and 97.50% and 100.00% with 4 mg.

**Fig. 5:**
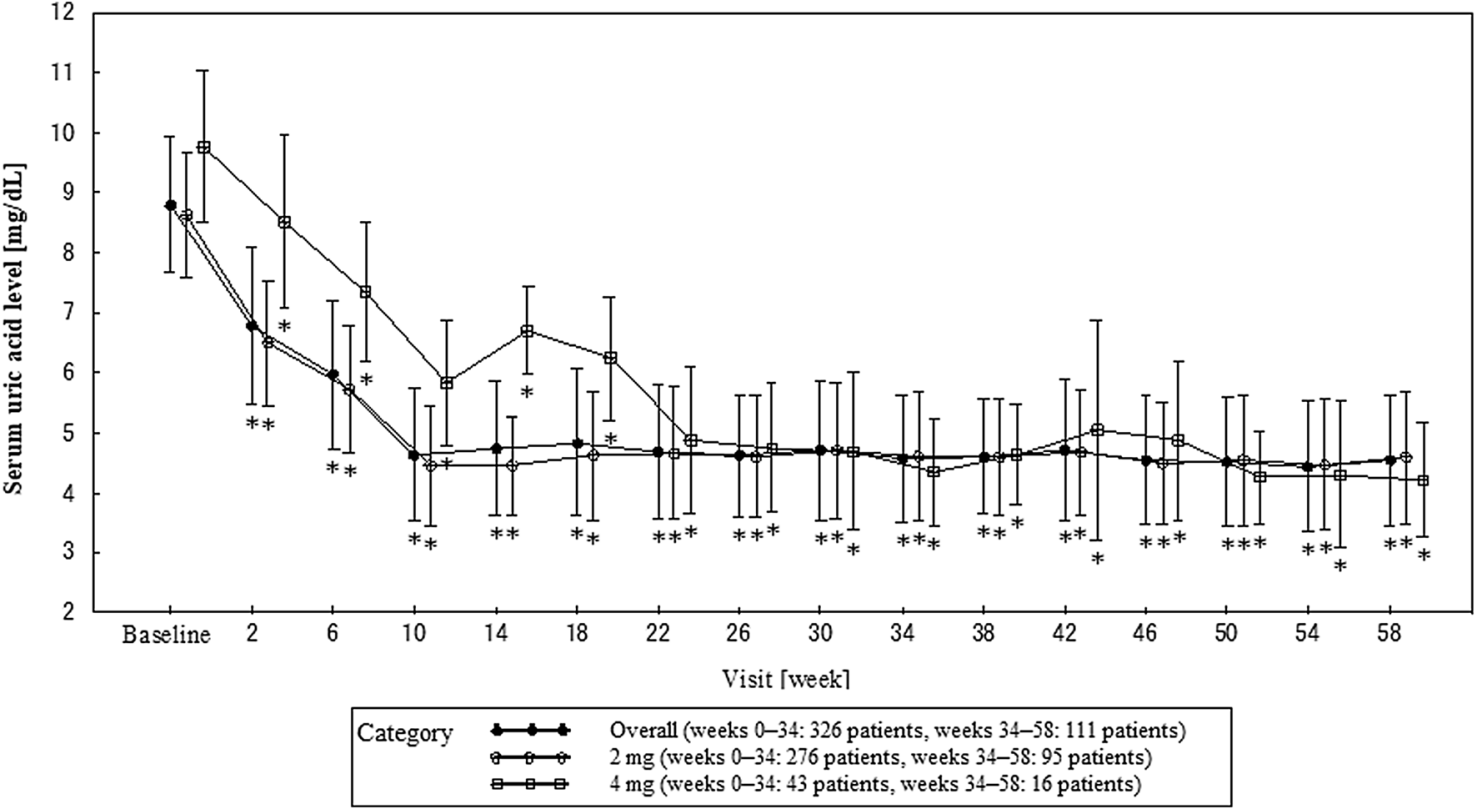
Changes in serum uric acid level in response to follow treatment with dotinurad. Error bars indicates standard deviation.*P < 0.05

Figure 5 shows the time-course of the mean serum uric acid levels at each visit. The mean serum uric acid levels at weeks 34 and 58 were as follows: 4.57 and 4.55 mg/dL overall; 4.61 and 4.59 mg/dL with 2 mg, and 4.35 and 4.22 mg/dL with 4 mg.

Table 3 shows the percent change in serum uric acid level from the baseline by sub-group, according to eGFR at week 34 and 58 (≥ 90, ≥ 60 to < 90, and ≥ 30 to < 60 mL/min/1.73 m^2^). The percent change in serum uric acid level was not meaningfully changed by eGFR.

**Table 3:** Sub-group analysis of percent change in serum uric acid levels at week 34 and 58 by category of eGFR at the baseline

eGFR^a^ category	Visit	Overall					2 mg					4 mg				
		*n*	Mean	±	SD	95% CI	*n*	Mean	±	SD	95% CI	*n*	Mean	±	SD	95% CI
Moderate	Week 34	65	47.48	±	9.81	45.05–49.92	58	46.61	±	9.40	44.14–49.08	7	54.73	±	10.93	44.62–64.83
	Week 58	19	48.57	±	8.37	44.54–52.60	19	48.57	±	8.37	44.54–52.60	0	–	±	–	–
Mild	Week 34	210	48.18	±	10.59	46.74–49.63	181	47.03	±	10.52	45.49–48.57	29	55.39	±	8.03	52.33–58.44
	Week 58	77	48.30	±	12.33	45.50–51.10	65	46.64	±	12.19	43.62–49.66	12	57.24	±	9.11	51.45–63.03
Normal	Week 34	22	45.52	±	15.59	38.61–52.44	18	44.11	±	16.45	35.93–52.29	4	51.90	±	10.10	35.82–67.97
	Week 58	9	49.33	±	8.93	42.46–56.20	8	48.17	±	8.80	40.82–55.53	1	58.59	±	–	–

eGFR for males (mL/min/1.73 m^2^) = 194 × Serum creatinine^−1.094^ × Age^−0.287^

eGFR for females (mL/min/1.73 m^2^) = 194 × Serum creatinine^−1.094^ × Age^−0.287^ × 0.739

*eGFR* (mL/min/1.73 m^2^) estimated glomerular filtration rate, *CI* confidence interval

^a^eGFR category, normal: eGFR ≥ 90 mL/min/1.73 m^2^, mild: eGFR ≥ 60 to < 90 mL/min/1.73 m^2^, moderate: eGFR ≥ 30 to < 60 mL/min/1.73 m^2^

The change in eGFR from the baseline to the final visit (mean ± SD) was 0.7 ± 6.8 mL/min/1.73 m^2^ overall, 0.9 ± 6.7 mL/min/1.73 m^2^ with 2 mg, and − 0.5 ± 7.6 mL/min/1.73 m^2^ with 4 mg (Table 4). Significant increase from the baseline was observed in the 2-mg group (paired *t *test: *P* = 0.038). The change in HOMA-IR from the baseline to the final visit (mean ± SD) was 0.13 ± 1.75 overall, 0.18 ± 1.63 with 2 mg, and − 0.17 ± 2.37 with 4 mg. No significant difference from the baseline was observed (paired *t *test: *P* > 0.05).

**Table 4:** The change in eGFR at week 34, week 58, and at the final visit

	Overall			2 mg			4 mg		
	*n*	Mean ± SD	*P* value	*n*	Mean ± SD	*P* value	*n*	Mean ± SD	*P* value
eGFR value (mL/min/1.73 m^2^)									
Baseline	326	69.6 ± 13.2	–	276	69.7 ± 12.7	–	43	70.0 ± 15.7	–
Week 34	299	70.1 ± 13.8	–	259	70.1 ± 13.5	–	40	70.4 ± 15.6	–
Week 58	105	69.0 ± 14.2	–	92	69.0 ± 14.6	–	13	68.4 ± 10.7	–
Final visit	310	70.2 ± 14.3	–	265	70.4 ± 14.0	–	41	70.1 ± 15.5	–
Change in eGFR (mL/min/1.73 m^2^)									
Week 34	299	0.6 ± 6.2	0.122	259	0.6 ± 6.0	0.081	40	− 0.1 ± 7.3	0.949
Week 58	105	− 1.1 ± 7.8	0.158	92	− 0.7 ± 7.8	0.416	13	− 4.1 ± 7.7	0.081
Final visit	310	0.7 ± 6.8	0.089	265	0.9 ± 6.7	0.038*	41	− 0.5 ± 7.6	0.684

*P < 0.05 (vs. baseline)

**Table 5:** Summary of AEs

	Overall		2 mg*		4 mg*	
	(*n* = 330)		(*n* = 277)		(*n* = 43)	
	Number of patients	Incidence (%)	Number of patients	Incidence (%)	Number of patients	Incidence (%)
AE	215	65.2	179	64.6	30	69.8
ADR	72	21.8	52	18.8	15	34.9
Serious AE	9	2.7	8	2.9	1	2.3
Serious ADR	1	0.3	1	0.4	0	0.0
AE leading to death	0	0.0	0	0.0	0	0.0
ADR leading to death	0	0.0	0	0.0	0	0.0
AE leading to discontinuation	15	4.5	8	2.9	3	7.0
ADR leading to discontinuation	10	3.0	3	1.1	3	7.0
AEs with incidence ≥ 5%						
Nasopharyngitis	59	17.9	49	17.7	10	23.3
Gouty arthritis	43	13.0	31	11.2	9	20.9

Dictionary for terms: MedDRA Ver. 21.0

*AE* adverse event, *ADR* adverse drug reaction

*Dose at completion of treatment (excluding patients who dropped out at a dose of 0.5 or 1 mg)

### Safety

Table 5 summarizes AEs in this study. The incidence of AEs was 65.2% overall, 64.6% with 2 mg, and 69.8% with 4 mg. The incidence of ADRs was 21.8% overall, 18.8% with 2 mg, and 34.9% with 4 mg. AEs reported in ≥ 5% patients overall were nasopharyngitis (17.9%) and gouty arthritis (13.0%). The severity of these AEs was mild or moderate.

The overall incidence of serious AEs was 2.7% and a causal relationship with dotinurad was not excluded for gastric cancer stage I alone among all of the serious AEs (gastrointestinal stromal tumor, bladder cancer, radius fracture, ameloblastoma, angina, acute cholecystitis, chronic sinusitis, gastric cancer stage I, and diverticulitis). No deaths were reported. The incidence of gouty arthritis was 13.0% at the completion of treatment across all doses and the severity was mild or moderate for all events.

The incidence of gouty arthritis was 1.2% during initial period I and 2.4% during initial period II. The incidence remained at ≤ 1.0% from week 34 to week 58 on the maintenance dose of dotinurad (Table 6). Additionally, the incidence of gouty arthritis at the final visit was 11.2% with 2 mg and 20.9% with 4 mg. The incidence tended to be higher at the 4 mg dose, based on the incidence when dotinurad was administered to all subjects similarly, using the same dosage regimen (initial period I: 0.5 mg, initial period II: 1 mg, maintenance period: 2 mg) before week 18, and no major difference in the incidence of gouty arthritis after week 18 was found between subjects who required dose escalation to 4 mg and those who continued with 2 mg after week 18.

**Table 6:** Number of patients and incidence by timing of gouty arthritis

Timing of dose		Overall			2 mg			4 mg		
		*n*	Number of patients	Incidence (%)	*n*	Number of patients	Incidence (%)	*n*	Number of patients	Incidence (%)
	Overall	330	43	13.0	277	31	11.2	43	9	20.9
Initial period I	Week 0–2	330	4	1.2	277	1	0.4	43	2	4.7
Initial period II	Week 2–6	328	8	2.4	277	3	1.1	43	3	7.0
Maintenance period I	Week 6–10	320	11	3.4	277	10	3.6	43	1	2.3
	Week 10–14	317	4	1.3	274	1	0.4	43	3	7.0
	Week 14–18	315	7	2.2	272	7	2.6	43	0	0.0
Maintenance period II	Week 18–22	313	12	3.8	270	10	3.7	43	2	4.7
	Week 22–26	308	5	1.6	265	5	1.9	43	0	0.0
	Week 26–30	305	1	0.3	263	1	0.4	42	0	0.0
	Week 30–34	303	3	1.0	262	2	0.8	41	1	2.4
	Week 34–38	108	0	0.0	94	0	0.0	14	0	0.0
	Week 38–42	107	0	0.0	93	0	0.0	14	0	0.0
	Week 42–46	107	1	0.9	93	1	1.1	14	0	0.0
	Week 46–50	106	0	0.0	92	0	0.0	14	0	0.0
	Week 50–54	105	1	1.0	92	1	1.1	13	0	0.0
	Week 54–58	105	1	1.0	92	1	1.1	13	0	0.0

Table 7 shows the changes in hepatic parameters (AST, ALT, γ-GTP) in this study. Although at certain points, AST and γ-GTP were significantly different from the baseline, these changes were within the range of normal physiological fluctuation and deviations from the reference value were slight.

**Table 7:** Summary of hepatic parameters

	Standard range	Visit	Overall			2 mg			4 mg		
			*n*	Mean ± SD	*P* value	*n*	Mean ± SD	*P* value	*n*	Mean ± SD	*P* value
AST (U/L)	10–40	Baseline	330	26.9 ± 9.0	–	277	26.2 ± 8.3	–	43	29.4 ± 9.6	–
		Week 34	300	28.0 ± 12.1	0.046*	260	27.4 ± 11.3	0.083	40	32.3 ± 16.2	0.329
		Week 58	105	28.6 ± 13.4	0.593	92	28.0 ± 13.0	0.592	13	32.4 ± 15.9	0.905
ALT (U/L)	5–45	Baseline	330	30.1 ± 15.5	–	277	29.3 ± 15.2	–	43	34.1 ± 15.5	–
		Week 34	300	31.9 ± 21.1	0.053	260	30.7 ± 19.5	0.173	40	39.8 ± 28.7	0.162
		Week 58	105	31.7 ± 19.7	0.904	92	31.3 ± 19.6	0.851	13	34.7 ± 20.8	0.909
γ-GTP (U/L)	Male ≤ 79 Female ≤ 48	Baseline	330	61.1 ± 44.1	–	277	59.6 ± 42.8	–	43	68.8 ± 47.9	–
		Week 34	300	67.0 ± 55.6	0.005*	260	64.7 ± 54.2	0.032*	40	82.0 ± 62.5	0.012*
		Week 58	105	60.7 ± 43.0	0.476	92	59.3 ± 39.8	0.317	13	70.7 ± 62.7	0.487

Paired *t *test was used to compare with baseline values

*AST* aspartate aminotransferase, *ALT* alanine aminotransferase, *γ-GTP* γ-glutamyl transpeptidase

*P < 0.05 (vs. baseline)

The incidence of renal calculi was 1.5% and all events occurred at week 34; no further events had occurred by week 58. Although all renal calculi were considered ADRs, no events were considered serious, and none required treatment.

## Discussion

In this study, dotinurad was administered at an initial dose of 0.5 mg/day for 2 weeks followed by 1 mg/day for 4 weeks and 2–4 mg/day as a maintenance dose for 28 or 52 weeks, to examine efficacy and safety in patients with hyperuricemia with or without gout.

At all visits, serum uric acid levels changed significantly from the baseline. Serum uric acid levels gradually decreased from week 2 and then remained stable at ≤ 6.0 mg/dL after administration of the maintenance dose (week 10). In addition, the serum uric acid lowering effect was not reduced with long-term administration. With 2-mg dose, the percentages of patients achieving a serum uric acid level ≤ 6.0 mg/dL at week 34 and 58 were 89.11% and 91.30%, respectively; with 4 mg, this rose to 97.50% and 100.00%, respectively. Of the 43 patients who failed to achieve a serum uric acid level ≤ 6.0 mg/dL on treatment with dotinurad 2 mg, 41 patients whose dose was increased to 4 mg achieved the target serum uric acid level. Although a renoprotective effect of XOIs has been reported [9, 10], its mechanism is unclear; a few reports describe the effects of increased urinary uric acid excretion. In a 6-month Phase III study with lesinurad, serious renal AEs were reported to occur in 4.7% of those on 400 mg monotherapy [5]. In the present study, no noteworthy changes with respect to renal impairment and renal parameters were seen with dotinurad. Moreover, eGFR at the final visit had significantly increased from the baseline with long-term treatment on dotinurad 2 mg. Table 4 shows eGFR and changes from the baseline to week 34 and 58 and the final visit. In consequence, we anticipate that studies examining the possibility that dotinurad might suppress renal impairment will be conducted in the future.

The incidence of AEs and ADRs were 65.2% and 21.8% overall, respectively; these did not increase with prolonged treatment. Additionally, when subjects who required dose escalation to 4 mg were compared to those in whom 2 mg dose was maintained, no meaningful changes in the incidence of AEs and ADRs were noted at each visit after week 18.

The Japanese management guidelines state that the frequency of gouty arthritis is decreased by maintaining a serum uric acid level ≤ 6.0 mg/dL using antihyperuricemic drugs [1]. In this study, the incidence of gouty arthritis from week 34 to 58 was ≤ 1.0% and tended to be lower in the latter half of the treatment period. Almost all patients achieved a serum uric acid level ≤ 6.0 mg/dL with long-term dotinurad treatment. Therefore, we assumed that continuous administration of dotinurad did suppress the onset of gouty arthritis.

In this study, renal calculi were found in five patients (1.5%). Although these events were considered ADRs, all were non-serious and no required treatment. With other antihyperuricemic drugs including XOIs, the onset of urinary calculi has also been reported. In a long-term study of lesinurad, kidney stones were reported in 2.0, 1.0, and 2.5% on allopurinol monotherapy, lesinurad 200 mg plus allopurinol, and lesinurad 400 mg plus allopurinol, respectively [11]. In the CRYSTAL study, nephrolithiasis was reported in 3.7, 0.9, and 1.8% in the febuxostat 80 mg, lesinurad 200 mg plus febuxostat, and lesinurad 400 mg plus febuxostat groups, respectively [12]. Although dotinurad increases urinary uric acid excretion, the incidence of urinary calculi was comparable to that seen with XOIs monotherapy. These reports constitute evidence that contributory factors for renal calculus formation include not just increased uric acid excretion in the urine, but also environmental factors like diet and lifestyle.

Fulminant hepatitis has been reported with benzbromarone, which is used mainly in Japan as a uricosuric drug. Furthermore, use of the benzbromarone is contraindicated in patients with hepatic impairment because of the risk of worsening hepatic impairment and regular hepatic function testing must be performed after starting administration. Additionally, all XOIs are associated with hepatic impairment as a significant ADR and they should be carefully administered to patients with hepatic dysfunction. In the present study, no clinically relevant changes in hepatic parameters were found with long-term dotinurad treatment. In addition, a clinical pharmacology study reported no major differences in pharmacokinetic parameters between subjects with mild-to-severe hepatic dysfunction and subjects with normal hepatic function and no safety concerns were observed [NCT03306667]. These results also support the contention that dotinurad is less likely than other antihyperuricemic agents to cause hepatic impairment.

The results of this study verified the efficacy and safety of dotinurad treatment for 58 weeks. At a dose of 2–4 mg, dotinurad adequately lowers serum uric acid in hyperuricemic patients with or without gout; this effect was maintained during long-term treatment, with a decreased onset of gouty arthritis.
